# Baicalein and Berberine Inhibit the Growth and Virulence of *Clostridioides difficile*

**DOI:** 10.3390/pathogens14070662

**Published:** 2025-07-04

**Authors:** Xue Yang, Dongming Zheng, Jiangyan Yong, Yuchen Li, Yunzhi Sun, Fei Zhao, Daiyan Tang, Yi Xie, Dongming Bi

**Affiliations:** 1 Department of Laboratory Medicine, Hospital of Chengdu University of Traditional Chinese Medicine, Chengdu 610072, China; xueyangxy123@gmail.com (X.Y.); jiangyanyong1@gmail.com (J.Y.); yuchenli1@outlook.com (Y.L.); yunzhisun322@gmail.com (Y.S.); 2 Department of Nuclear Medicine, Ya’an People’s Hospital, Ya’an 625000, China; zhendongming@stu.cdutcm.edu.cn; 3 Department of Laboratory Medicine, Deyang Hospital Affiliated Hospital of Chengdu University of Traditional Chinese Medicine, Deyang 618000, China; feizhao9@outlook.com; 4 College of Medical Technology, Chengdu University of Traditional Chinese Medicine, Chengdu 611137, China; tangdaiyan@stu.cdutcm.edu.cn; 5 Laboratory of Clinical Microbiology, Department of Laboratory Medicine, West China Hospital, Sichuan University, Chengdu 610041, China

**Keywords:** baicalein, berberine, *Clostridioides difficile*, toxins, antibiotics

## Abstract

*Clostridioides difficile* is a leading pathogen involved in healthcare-associated diarrhea. With its increasing incidence, mortality, and antibiotic resistance, there is an urgent need for novel therapeutic strategies to address the infection and prevent its recurrence. Gegen Qinlian Decoction (GQD) is a traditional Chinese medicine for the treatment of diarrhea, but its main active ingredient is not known. Therefore, in this study, we evaluated the biological activity of berberine (BER) and baicalein (BAI), key components of GQD, against *C. difficile*. Time–kill curves and scanning electron microscopy were employed to assess their effects on *C. difficile* growth, while Enzyme-Linked Immunosorbnent Assay (ELISA) and cytotoxicity assays were used to examine their impact on toxin production. We also employed Quantitative Reverse Transcription PCR (qRT-PCR) to examine how BER and BAI influenced the expression of toxin-associated genes. At sub-inhibitory concentrations, these compounds exerted antibacterial activity against *C. difficile* by disrupting the integrity of the cell membrane and cell wall. Furthermore, BER and BAI also suppressed toxin production, demonstrating effects comparable to those of vancomycin. This suppression likely resulted from their bactericidal activity and the inhibition of toxin gene expression. This study not only highlights the potential application of GQD in treating *C. difficile* infections but also offers promising options for developing drugs targeting the growth and virulence of this pathogen. *C. difficile* infection (CDI) is a leading cause of severe diarrhea, and its treatment remains challenging due to limited drug options and its high recurrence rate. BAI and BER, the main active components of the traditional Chinese medicinal formula GQD, inhibited the growth of *C. difficile* by disrupting its cellular structure and significantly reduced the production of toxins associated with disease severity. Furthermore, the effects of BAI and BER on *C. difficile* were comparable to those of conventional antibiotics, suggesting that these compounds could be potential alternative therapies for CDI. This study not only highlights the therapeutic potential of GQD in treating CDI but also provides a replicable research strategy for the development of novel anti-CDI agents.

## 1. Introduction

*Clostridioides difficile* is a Gram-positive, anaerobic, spore-forming bacterium with high infectivity and is a leading cause of healthcare-associated diarrhea worldwide [1,2]. *C. difficile infection* (CDI) can manifest with a range of clinical outcomes, from asymptomatic carriage to life-threatening conditions such as sepsis and toxic megacolon [3]. Although *C. difficile* primarily affects long-term-hospitalized patients or elderly individuals receiving long-term antibiotic therapy, the incidence of infection in the general population is rising [4]. It is estimated that approximately 41% of cases are now community-acquired, with an incidence rate among children reaching as high as 25.8 per 100,000 [5]. Notably, in 2013, the US Centers for Disease Control and Prevention classified *C. difficile* as an urgent antimicrobial resistance threat [6]. Vancomycin (VAN) and fidaxomicin are the first-line treatments for CDI. Recent studies conducted in both the United States and China have shown that *C. difficile* is highly susceptible to VAN and fidaxomicin, with only 0.7% of clinical isolates exhibiting resistance to VAN [7,8]. Despite the low overall resistance rate, the reduced susceptibility of *C. difficile* to VAN has still been linked to poorer treatment outcomes, such as decreased 30-day sustained clinical response and lower 14-day initial cure rates [9]. The clinical symptoms of CDI are primarily attributed to toxins A (TcdA) and B (TcdB), which are encoded by the genes *tcdA* and *tcdB*, respectively. These toxins damage intestinal epithelial cells, disrupt the mucosal barrier, and trigger a strong inflammatory response [10]. The expression of *tcdA* and *tcdB* is positively regulated by *tcdR* and negatively regulated by *tcdC*; the latter regulates toxin expression by regulating *tcdR*. Additionally, the holin-like protein encoded by *tcdE* is essential for toxin secretion [11]. Against this backdrop, the development and discovery of novel antibiotics targeting *C. difficile* growth and virulence could present new therapeutic strategies to combat CDI.

For centuries, plants with ethnopharmacological significance have been a rich source of medicinal compounds, many of which exhibit remarkable biological activities, including antidiarrheal effects [12]. Recent studies have found that *Galla chinensis* and its major component, tannic acid, can inhibit toxin production and spore formation by *C. difficile* in vitro, and *Galla chinensis* has been used to treat diarrhea, dysentery, hemorrhoids, and other related conditions [13,14]. Gegen Qinlian Decoction (GQD) is a traditional Chinese medicine (TCM) prescription composed of *Pueraria lobata*, *Glycyrrhiza uralensis*, *Coptis chinensis*, and *Scutellaria baicalensis* and is an effective remedy for the treatment of acute diarrhea [15]. GQD exhibits a wide range of pharmacological effects, including lipid-lowering, antibacterial, antiviral, and gastrointestinal function-regulating activities [16]. Additionally, GQD can treat ulcerative colitis by modulating the gut microbiota [17]. Although berberine (BER), the main component of *Coptis chinensis*, has been shown to restore gut microbiota imbalances caused by VAN treatment, thus preventing CDI recurrence [18], the specific active ingredients responsible for the key antibacterial effects of GQD and whether they can reduce *C. difficile* toxin secretion remain unclear. Therefore, we selected this traditional Chinese herbal formula, commonly used to treat CDI-associated diarrhea, to investigate the effects of its constituent herbs and their major active components on the growth and virulence of *C. difficile* in vitro. This study not only provides scientific support for the potential application of GQD in CDI treatment but also contributes to ongoing efforts in the discovery of novel antimicrobial agents. By screening ethnopharmacological plants with potential anti-CDI properties (such as those highlighted in traditional Chinese formulations summarized by Gao et al. [15]) and analyzing their bioactive components, our findings may facilitate further pharmacological research and accelerate the development of new therapeutic strategies against *C. difficile*.

## 2. Materials and Methods

### 2.1. Strains and Media

The *C. difficile* standard strain ATCC 9689, provided by the Shanghai Bioresource Collection Center, was stored in 50% glycerol at −80 °C. This strain belongs to ribotype 001 and is known to produce both TcdA and TcdB [19]. Before use, it was thawed at room temperature, inoculated into a semi-fluid trypsin-digested meat medium, and passaged twice. A single colony was subsequently inoculated into brain–heart infusion (BHI) medium and incubated anaerobically at 37 °C for 24 h. The bacterial suspension was then adjusted to 1 × 10^6^ CFU/mL for further experiments.

### 2.2. Tested Substances

The herbal medicine *Galla chinensis* and the constituent herbs of the GQD formula—*Pueraria lobata*, *Glycyrrhiza uralensis*, *Coptis chinensis*, and *Scutellaria baicalensis*—were replaced with Decoction-Free Chinese Herbal Granules (DCHGs) instead of the traditional decoction. These were purchased from Sichuan Neautus TCM Co., Ltd (Chengdu, China). The DCHGs were prepared by purifying and concentrating single herbal ingredients, offering the same therapeutic efficacy as traditional decoctions [20]. The active ingredients of these herbs, including BER, baicalein (BAI), baicalin, and tannic acid, were purchased from Shanghai Macklin Biochemical Technology Co., Ltd (Shanghai, China). The DCHGs were dissolved in BHI medium at an initial concentration of 40,960 μg/mL, while the initial concentrations of BER, BAI, baicalin, and tannic acid were 4096 μg/mL. Additionally, VAN (Beyotime, shanghai, China; Product No. Y256394) was used as an antibiotic control group to compare the inhibitory effects of the aforementioned drugs on the growth and virulence of *C. difficile*.

### 2.3. Detection of the Minimum Inhibitory Concentration (MIC)

The MIC of substances tested against *C. difficile* was determined using the broth dilution method [21,22]. Briefly, these tested substances were serially diluted in BHI medium in sterile test tubes, creating 10 concentration gradients. Subsequently, 100 μL of the tested substance solution and 100 μL of bacterial suspension were added to a sterile 96-well plate to achieve a final bacterial concentration of 5 × 10^5^ CFU/mL. At the same time, a blank group (containing only culture medium) and an untreated group (containing only bacterial suspension) were set up as controls. After anaerobic incubation at 37 °C for 48 h, the MIC was defined as the lowest concentration of the compound that inhibited visible bacterial growth. Additionally, agar was added to the BHI medium to further confirm the MIC.

### 2.4. Checkerboard Dilution Experiment

A checkerboard dilution assay was used to test the synergistic effect of BER and BAI on *C. difficile* [23,24]. Different concentrations of BER and BAI were applied to BHI agar plates to create combinations of double the MIC, the MIC, 1/2 MIC, and 1/4 MIC for both compounds. *C. difficile* was then inoculated onto the plates, followed by anaerobic incubation. After 48 h of incubation, bacterial growth was visually assessed to determine the synergistic interaction pattern.

### 2.5. Time–Kill Curve Assay

Time–kill curve analysis was performed to monitor the dynamic antibacterial effects of sub-inhibitory concentrations of BER and BAI against *C. difficile* [25]. *C. difficile* was adjusted to a concentration of 1 × 10^6^ CFU/mL in BHI liquid medium. The bacteria were then treated with BER and BAI at 1/4 MIC and 1/2 MIC, as well as 4 μg/mL of VAN, and incubated anaerobically at 37 °C. OD_600_ readings were taken at 0, 6, 12, 24, 36, 48, and 72 h to measure bacterial growth, with all measurements repeated three times to construct the growth curve.

### 2.6. Scanning Electron Microscopy (SEM)

The ultrastructural effects of BER and BAI on *C. difficile* were observed using SEM [26]. After co-incubating *C. difficile* with BER and BAI for 12 and 24 h, bacterial pellets were collected. The cells were washed three times with sterile PBS and fixed at room temperature for 2 h using an electron microscope fixation solution. Then, the samples were fixed with 1% osmium tetroxide for 1 h, followed by dehydration through a series of ethanol gradients. Finally, the samples were transferred to silicon wafers and coated using an ion sputter. The specimens were visualized using a Hitachi 2500 SEM (Hitachi, Tokyo, Japan).

### 2.7. Enzyme-Linked Immunosorbnent Assay (ELISA)

The levels of TcdA and TcdB released by *C. difficile* after treatment with BER and BAI were measured using the *C. difficile* TcdA/TcdB ELISA kit (Jingmei, Yancheng, China; Product No. JM-00173O1/JM-00174O1). The overnight-cultured *C. difficile* was diluted in BHI medium to 1 × 10^6^ CFU/mL, followed by drug addition and anaerobic incubation at 37 °C for 6, 12, 24, and 48 h. The bacterial supernatant was collected using centrifugation. According to the manufacturer’s instructions, 50 μL of standards, sample diluent, or supernatant was added to the ELISA plate wells, forming standard, zero, and sample wells, while blank wells received no additions. Then, 100 μL of horseradish peroxidase-conjugated detection antibody (except in blank wells) was added, and the plate was incubated at 37 °C in the dark for 60 min. After washing, 50 μL each of substrate solutions A and B were added and incubated at 37 °C for 10 min. The reaction was stopped with 50 μL of stop solution, and absorbance was measured at 450 nm within 15 min.

### 2.8. Vero Cell Cytotoxicity Assay

As previously mentioned, the Vero cell cytotoxicity assay was used to assess the effects of BER and BAI on the cytotoxicity of *C. difficile* culture supernatants [27]. After 24 h of treatment with BER and BAI, the supernatants from *C. difficile* cultures were collected and serially diluted 4-fold. The diluted supernatants were then added to a monolayer of Vero cells in a 96-well plate. The plates were incubated at 37 °C for 24 h and examined under an inverted microscope. Positive reactions were indicated by the characteristic rounding of Vero cells. A neutralization assay was simultaneously performed using *C. difficile* antitoxin antibodies (Otwo, Guangzhou, China) to verify the specificity of the observed cytotoxicity.

### 2.9. Quantitative Reverse Transcription PCR (qRT-PCR)

The effects of BER and BAI on the expression of *C. difficile* toxin synthesis genes were analyzed using qRT-PCR. After 24 h of treatment with the drugs, *C. difficile* cells were collected using centrifugation at 3000× *g* for 10 min at 4 °C. The cells were rapidly frozen in liquid nitrogen, and total RNA was extracted using TRIzol^®^ reagent (Invitrogen, Carlsbad, CA, USA). According to the manufacturer’s instructions, cDNA was synthesized using the BeyoRT™ II cDNA Synthesis Kit (Beyotime, Shanghai, China), and qPCR was performed with TransStart Green qPCR SuperMix (Transgen, Beijing, China). The primers for the target and reference (16S rRNA) genes were synthesized by Shanghai Biotech (Table 1). Relative gene expression was calculated using the 2^−ΔΔCt^ method.

### 2.10. Statistical Analysis

All experiments were conducted in triplicate under identical conditions. Data were analyzed using SPSS 26.0 software, and results were expressed as the mean ± standard deviation [28]. Statistical differences was evaluated according to previously published methods using ANOVA followed by Tukey’s HSD test [29]. Statistical significance was considered present at *p <* 0.05.

## 3. Results

### 3.1. Antibacterial Activity of Tested Substances Against C. difficile

The results of the susceptibility testing are shown in Table 2. Among the tested Decoction-Free Chinese Herbal Granules, *Galla chinensis* exhibited the lowest MIC of 64 μg/mL against *C. difficile*, followed by *Coptis chinensis*, with an MIC value of 1024 μg/mL. However, *Pueraria lobata*, *Glycyrrhiza uralensis*, and *Scutellaria baicalensis* showed no inhibitory effect against *C. difficile*. In contrast, the active compounds BER and BAI from *Coptis chinensis* and *Scutellaria baicalensis* demonstrated strong antibacterial activity, with MIC values of 256 and 320 μg/mL, respectively. In comparison, the active component tannic acid from *Galla chinensis* exhibited weaker inhibitory effects. Due to the high sensitivity of *C. difficile* to BER and BAI, we further investigated the effects of sub-MICs (1/4 MIC and 1/2 MIC) of BER and BAI on the growth and virulence of this pathogen in subsequent experiments.

### 3.2. Time–Kill Curves of BER and BAI Against C. difficile

**Figure 1:**
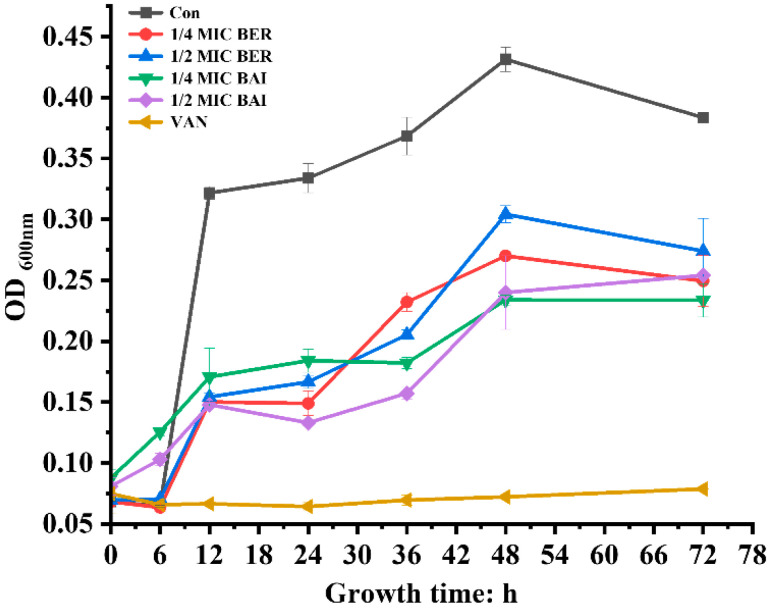
Killing kinetics of *C. difficile*. After the treatment of ATCC 9689 with Berberine (BER) at 1/4 MIC (64 μg/mL) and 1/2 MIC (128 μg/mL), Baicalein (BAI) at 1/4 MIC (80 μg/mL) and 1/2 MIC (160 μg/mL), and Vancomycin (VAN) at 1 MIC (4 μg/mL), optical density (OD) was measured at 0, 6, 12, 24, 36, 48, and 72 h. Three independent experiments were performed. Values represent the mean ± standard deviation.

We first investigated the dynamic antibacterial effects of sub-MICs of BER and BAI through a 72 h killing kinetics experiment (Figure 1). Compared to that in the control group, growth in all other groups was significantly delayed. The strongest inhibitory effects of BER and BAI were observed at 12 h. However, the inhibitory effects of both compounds weakened over time after this point, and no significant differences were observed between the various concentrations. Notably, VAN at 4 μg/mL completely inhibited *C. difficile* growth from the start. This suggests that at sub-inhibitory concentrations, BER and BAI only slow down the proliferation and growth of *C. difficile* without exhibiting bacteriostatic activity. Subsequently, we investigated the Fractional Inhibition Concentration Index (FICI) of BER and BAI and found that it was 2 for the combination of them. This indicates that these compounds do not exhibit a synergistic effect on *C. difficile*, and the inhibition effect is primarily dependent on the action of each drug.

### 3.3. Effects of BER and BAI on the Cell Structure of C. difficile

**Figure 2:**
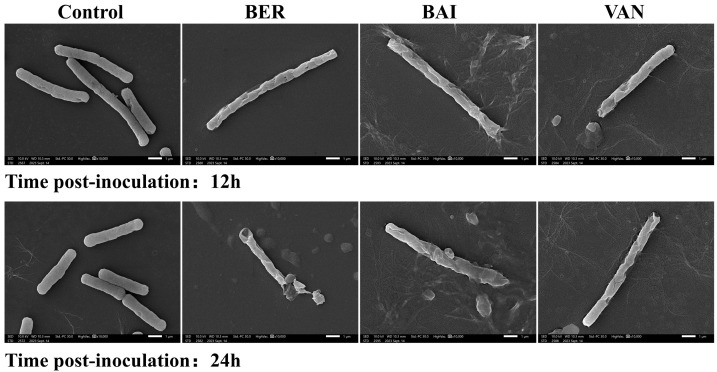
BER and BAI disrupted the cell membrane and cell wall (magnification: ×10,000).

To further investigate the bactericidal effects of BER and BAI on *C. difficile*, we observed the morphological changes in the bacteria (Figure 2). In the control group, *C. difficile* exhibited a typical rod-shaped structure with a smooth surface, intact cell walls, and clearly visible intracellular septa. However, after 12 and 24 h of treatment with BER (1/2 MIC), BAI (1/2 MIC), and VAN (MIC), the bacterial surfaces showed significant wrinkling and indentation, and the intracellular septa were absent. The cell walls were damaged and collapsed, with the leakage of bacterial contents.

### 3.4. Effect of BER and BAI on C. difficile Toxin Production

**Figure 3:**
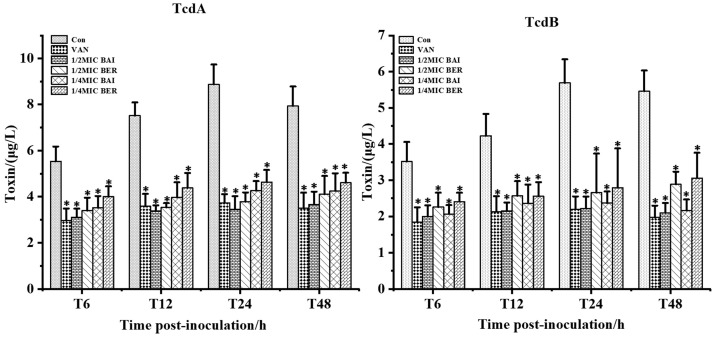
BER and BAI inhibit toxin production. * indicates a statistically significant difference compared to the control group (*p <* 0.05).

The effects of BER and BAI on *C. difficile* toxin production are shown in Figure 3. Compared to the control group, BER, BAI, and VAN significantly reduced the production of TcdA and TcdB at all time points (6 h, 12 h, 24 h, 48 h). At 24 h, the inhibition of toxin production was strongest, with BER (1/2 MIC), BAI (1/2 MIC), and VAN (MIC) achieving inhibition rates of 57%, 61%, and 58% for TcdA and 54%, 61%, and 61% for TcdB, respectively. Moreover, BER and BAI exerted similar inhibitory effects on toxin production. However, consistently with antibacterial activity, no significant difference was observed in the effects of different concentrations of BER and BAI on toxin production.

### 3.5. Effects of BER and BAI on C. difficile-Mediated Cytotoxicity

**Figure 4:**
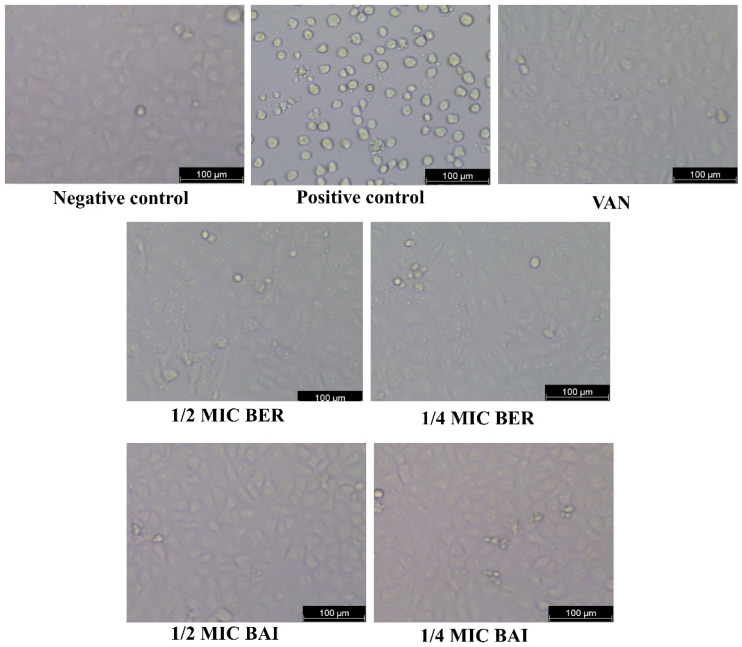
BER and BAI reduced cytotoxicity induced by *C. difficile* toxins (magnification: ×100).

To determine whether the inhibitory effects of BER and BAI on *C. difficile* toxin production would attenuate the impact on cell damage, we conducted a cytotoxicity assay. As shown in Figure 4, Vero cells with normal growth appeared elliptical, while untreated *C. difficile* supernatants exhibited a characteristic rounding effect. In contrast, exposure to BER, BAI, and VAN significantly reduced the cytotoxicity of these supernatants on Vero cells. Compared to the endpoint titer of 4^8^ in the untreated group, the endpoint titers of BER, BAI, and VAN were all 4^3^, representing a reduction of more than 90%.

### 3.6. Effects of BER and BAI on the Toxin Synthesis Genes of C. difficile

**Figure 5:**
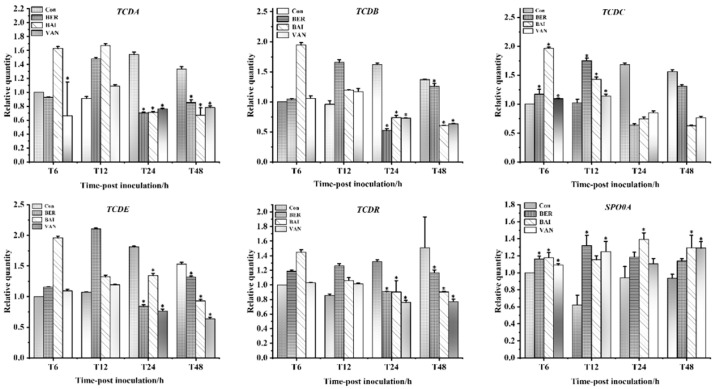
Inhibition of toxin synthesis gene expression by BER and BAI. * indicates a statistically significant difference compared to the control group (*p < 0.05*).

Next, we investigated whether the inhibitory effects of BER and BAI on *C. difficile* virulence are associated with changes in virulence gene expression (Figure 5). The results showed that BER (1/2 MIC), BAI (1/2 MIC), and VAN (MIC) effectively suppressed the expression of *tcdA* and *tcdB* only after 24 and 48 h of treatment. Specifically, at 24 h, BER and BAI reduced *tcdA* expression 2.21- and 2.16-fold, respectively, and *tcdA* expression 3.12- and 2.22-fold. Similarly, the expressions of the toxin secretion gene *tcdE* and the positive regulator *tcdR* were inhibited by both compounds at 24 and 48 h. Notably, at 6 and 12 h, both BER and BAI appeared to enhance the expression of *tcdA*, *tcdB*, *tcdE*, and *tcdR*. The effects on *tcdC*, which encodes an antagonist of TcdR, were opposite, with promotion observed at 6 and 12 h and suppression at 24 and 48 h. Additionally, we examined the expression of *SPO0A*, a key regulator of *C. difficile* sporulation. Although BER and BAI upregulated *SPO0A*, the increase was relatively modest.

## 4. Discussion

In recent years, the rising incidence of CDI has become a critical concern for public health and healthcare systems [30]. Simultaneously, the widespread use of antibiotics has led to an increase in *C. difficile* resistance, further limiting the already-restricted options for antibiotic treatment [31]. In light of this, we selected GQD, a TCM prescription known for its effectiveness in treating diarrhea, and investigated the effects of its individual herbal monomers and main active ingredients on *C. difficile* growth and virulence in vitro. The goal was to explore alternative options for CDI treatment. Our results indicate that *Coptis chinensis* is the primary herbal monomer responsible for inhibiting *C. difficile* growth in GQD. Further investigation revealed that even at sub-MICs, the main active components of *Coptis chinensis* (BER) and *Scutellaria baicalensis* (BAI) exhibited similar effects to those of VAN, effectively inhibiting *C. difficile* growth and toxin production.

*Coptis chinensis* exhibits antimicrobial activity against various pathogenic microorganisms, with reported MICs of 1095 μg/mL for *Staphylococcus aureus*, 5000 μg/mL for *Klebsiella pneumoniae*, 813 μg/mL for *Streptococcus pyogenes*, and 6250 μg/mL for *Candida albicans* [32,33,34,35]. Consistently with this, our study found that only *Coptis chinensis* in GQD exhibited antibacterial activity, with an MIC of 1024 μg/mL. In contrast, *Glycyrrhiza uralensis*, *Scutellaria baicalensis*, and *Pueraria lobata* did not inhibit *C. difficile* growth. To exclude the potential impact of pharmacological differences between DCHGs and traditional decoctions on the results, we also tested the MIC of *Galla chinensis*, and our result of 64 μg/mL closely aligned with that of Wang et al. [14](128 μg/mL). However, among the active ingredients of TCMs, we found that both BER and BAI effectively inhibited the growth of *C. difficile*, with MICs of 256 μg/mL and 320 μg/mL, respectively. Further, and significantly, it has been reported that the contents of BER and BAI in *Coptis chinensis* and *Scutellaria baicalensis* are approximately 10% and 0.1–1.6%, respectively. Although *Scutellaria baicalensis* does not exhibit antibacterial activity, recent studies have shown that BAI significantly inhibits highly pathogenic bacteria such as *Candida auris* and *Acinetobacter baumannii* [36,37]. Indeed, the combination of active components in TCMs exhibits complex interactions in antimicrobial activity. For instance, BER combined with eugenol and pterostilbene exhibits synergistic effects against fungi, reducing the MIC of BER by 8–16-fold. In contrast, β-escin, when combined with eugenol and curcumin, displays antagonistic effects [38]. Additionally, our study likewise demonstrates that the combination of BER and BAI does not result in synergistic antibacterial activity. This may explain why *Scutellaria baicalensis* alone cannot inhibit the growth of *C. difficile*, while its active components, baicalin and BAI, exhibit inhibitory effects. This also indicates that other active compounds present in these herbs may contribute to or modulate their overall therapeutic efficacy against *C. difficile*. Undoubtedly, BER (4–1000 μg/mL) exhibits significant antimicrobial activity against nearly all human pathogens, including bacteria, fungi, and viruses [39,40]. Our findings further confirm its antibacterial efficacy against *C. difficile*, suggesting that BER holds promise as a potential candidate for future antimicrobial therapies. However, compared with commonly used antibiotics such as VAN, BER and BAI exhibit higher MICs against *C. difficile*, indicating only moderate antibacterial activity. Furthermore, the potential of BER and BAI in the treatment of CDI needs to be evaluated in conjunction with their pharmacokinetic characteristics in feces. Previous studies have shown that the oral administration of vancomycin (250 mg, four times daily) can maintain fecal concentrations exceeding 2000 µg/mL. This level is significantly higher than the EUCAST-defined resistance breakpoint (MIC > 2 µg/mL) [41,42]. However, no direct pharmacokinetic data regarding the fecal concentrations of BER and BAI are currently available. Therefore, future studies should focus on structural optimization, the determination of effective in vivo dosages (including those of both active compounds and crude herbal materials), and the development of nanoparticle-based delivery systems to enhance their antimicrobial activity, along with the systematic evaluation of their pharmacokinetics in feces, to support their potential application in CDI treatment.

It has been reported that the antimicrobial activity of BER is time-dependent [40]. However, we found that at 1/2 MIC and 1/4 MIC, the antibacterial activity of this compound and BAI against *C. difficile* peaked at 12 h but gradually diminished over time. In contrast, BER (at the MIC) completely inhibited the growth of *Escherichia coli* and *Bacillus subtilis* within 18 h in other research [43]. Additionally, recent studies have shown that the sub-MIC of BER has no effect on *C. difficile* biofilm formation [23]. This suggests that BER and BAI may only exert their time-dependent antibacterial effects at concentrations ≥ MIC, with the stronger inhibition of planktonic cells compared to biofilm cells. Our SEM results further support these findings. We observed that at 12 h, both BER and BAI caused significant damage to the cell membrane and cell wall of *C. difficile*. Previous studies have shown that BER disrupts the integrity of the *S. aureus* cell membrane and cell wall in a dose-dependent manner, leading to severe cell wall rupture and blurred boundaries [44]. Similarly, BAI inhibits *E. coli* proliferation by disrupting its cell wall [45]. Therefore, the inhibition of *C. difficile* growth by BER and BAI may be attributed to their effects on the cell membrane and cell wall. This also explains why the combination of BER and BAI does not exhibit synergistic effects, as compounds with similar mechanisms of action are unlikely to produce additive effects [38].

CDI is a toxin-mediated disease, and targeting toxin production is considered an effective strategy for its treatment. Recent studies have shown that treatment with actoxumab (anti-TcdA antibody) or bezlotoxumab (anti-TcdB antibody) significantly reduces CDI recurrence [46]. In a mouse model, the use of tannic acid significantly improved CDI survival rates by reducing serum TcdA levels [14]. Our findings indicate that sub-MICs of BER and BAI significantly reduced the production of TcdA and TcdB compared to the untreated control group. Consistently with this, cell experiments showed that the supernatants from *C. difficile* treated with BER and BAI exhibited significantly reduced cytotoxicity toward Vero cells. Similarly, Pellissery et al. [47] reported that baicalin decreased toxin production in *C. difficile*. Importantly, previous studies have demonstrated that BER and BAI alone significantly alleviate diarrhea and mortality caused by *C. difficile* in mouse models [18,45]. These findings indicate that the inhibition of toxin production by BER and BAI contributes to improving CDI symptoms in vivo. To investigate the mechanism underlying the reduction in toxin production, we assessed the expression of toxin-associated genes. Treatment with BER and BAI upregulated *tcdC* expression at 6 and 12 h. Although *tcdC* is often regarded as a negative regulator of toxin secretion, conflicting studies suggest that this gene may have no significant impact on toxin production [48]. Moreover, mathematical modeling has indicated that the loss of *tcdC* function is likely to contribute to increased toxin levels in hypervirulent strains only when *tcdC* is continuously expressed throughout the growth phase and produced at higher levels than *tcdR* [49]. However, in our study, BER and BAI significantly downregulated *tcdA*, *tcdB*, *tcdE*, and *tcdR* only at 24 and 48 h. These findings suggest that the reduced expression of these genes, rather than the upregulation of *tcdC*, may have been responsible for the observed decrease in toxin production and secretion. Notably, BER and BAI consistently inhibited toxin production at all time points. Based on the time–kill curve data, we hypothesize that the early reduction in toxin production (6–12 h) was primarily due to the suppression of *C. difficile* growth, whereas the later reduction (24–48 h) is mainly attributed to the inhibition of toxin gene expression. At the very least, over 6 and 12 h, the complete inhibition of *C. difficile* growth by VAN led to a reduction in its toxin levels. However, as we did not experimentally confirm whether BER and BAI can directly bind to toxins A and B to reduce the cytotoxicity of *C. difficile*, the possibility that they exert their effects by directly neutralizing these toxins cannot be excluded. Moreover, no clear concentration-dependent effect of BER and BAI on bacterial growth or toxin secretion was observed. Although this may be partly attributed to the use of sub-MICs that are insufficient to produce strong inhibitory effects, whether higher concentrations could result in more pronounced suppression remains to be further investigated. In addition, spores are the primary form in which *C. difficile* exists in the external environment, and they are one of the main reasons for the widespread transmission of CDI [50]. Interestingly, we observed that BER and BAI upregulated *SPO0A* expression. Correspondingly, recent studies have reported that sub-MICs of BER do not affect *C. difficile* spore germination and impair the motility of *C. difficile*, and higher concentrations are not required to inhibit growth [23].

In summary, this study demonstrates that sub-MICs of BER and BAI inhibit the growth of *C. difficile* by disrupting its cell membrane and cell wall while also reducing toxin production through the downregulation of toxin synthesis genes. These findings not only identify new compounds with therapeutic potential for treating CDI but also support the millennia-old tradition of using GQD for treating acute diarrhea in China. However, Chinese herbal extracts typically contain multiple active components that target different pathways in the synergistic treatment of various diseases. While our study indicates that BER and BAI do not exhibit a synergistic effect, the presence of other compounds in GQD that might have such effects warrants further investigation. Currently, we are conducting a comprehensive analysis of the active ingredients in GQD to identify effective compound combinations for the in vivo treatment of CDI. Furthermore, given the significant differences in the genome, virulence factors, antibiotic resistance, and other biological characteristics between clinical isolates and standard strains, in this study, we only used a standard *C. difficile* strain. While this approach helped to preliminarily validate the antibacterial and antitoxic properties of BER and BAI, their effects on a broader range of clinical strains remain unclear. Therefore, future studies should focus on clinical *C. difficile* isolates, particularly the highly virulent ribotype 027 and 078 strains, as well as multidrug-resistant strains, to further validate the antibacterial and antitoxic effects of BER and BAI. Additionally, high-quality in vivo studies are needed to comprehensively assess these compounds’ clinical application potential, providing a stronger experimental foundation for future clinical treatments.

## Figures and Tables

**Table 1:** Primer sequences used in this study.

Oligo Name	Sequence (5′ to 3′)	GC (%)	TM (°C)	Product Size (bp)
16S RNA-F	AGCGGTGAAATGCGTAGATAT	42.86	57.61	72
16S RNA-R	CAGCGTCAGTTACAGTCCAGA	52.38	59.73	
*SPO0A*-F	TTTTAGCAGATGACAATAAGG	33.33	51.54	674
*SPO0A*-R	GTCAACTTTTCCTCTACTCCA	42.86	54.89	
*tcdA*-F	GCTTTCGCTTTAGGCAGTGT	50.00	59.12	126
*tcdA*-R	GGCTGGGTTAAGGTGTTGGT	55.00	60.18	
*tcdB*-F	ATGGAAGGTGGTTCAGGTCA	50.00	58.56	204
*tcdB*-R	ACCTGGTGTCCATCCTGTTTC	52.38	59.93	
*tcdC*-F	ACCATGGTTCAGCATCAGACA	47.62	59.65	171
*tcdC*-R	AGGGTATTGCTCTACTGGCA	50.00	58.12	
*tcdE*-F	ACCTAGGAGGCGTTATGAATATGAC	44.00	60.05	170
*tcdE*-R	TGCTACTTTTCTGATTCCTCCA	40.91	57.09	
*tcdR*-F	AACTCAGTAGATGATTTGCAAGAA	33.33	56.62	100
*tcdR*-R	TTAAATCTGTTTCTCCCTCTTCA	34.78	55.25	

**Table 2:** MICs of tested substances against *C. difficile*.

Category	Substance	MIC (μg/mL)
Decoction-Free Chinese Herbal Granules	*Galla chinensis*	64
	*Coptis chinensis*	1024
	*Glycyrrhiza uralensis*	>40,960
	*Scutellaria baicalensis*	>40,960
	*Pueraria lobata*	>40,960
Herbs	Berberine	256
	Baicalein	320
	Baicalin	512
	Tannic acid	2560
Antibiotics	Vancomycin	4

## Data Availability

The data that support the findings of this study are available from the corresponding author upon reasonable request.
